# Functional and structural studies on the *Neisseria gonorrhoeae* GmhA, the first enzyme in the *glycero‐manno*‐heptose biosynthesis pathways, demonstrate a critical role in lipooligosaccharide synthesis and gonococcal viability

**DOI:** 10.1002/mbo3.432

**Published:** 2017-01-07

**Authors:** Igor H. Wierzbicki, Ryszard A. Zielke, Konstantin V. Korotkov, Aleksandra E. Sikora

**Affiliations:** ^1^ Department of Pharmaceutical SciencesCollege of PharmacyOregon State UniversityCorvallisORUSA; ^2^ Department of Molecular & Cellular BiochemistryCollege of MedicineUniversity of KentuckyLexingtonKYUSA

**Keywords:** crystal structure, drug target, *Neisseria gonorrhoeae*, sedoheptulose‐7‐phosphate isomerase GmhA

## Abstract

Sedoheptulose‐7‐phosphate isomerase, GmhA, is the first enzyme in the biosynthesis of nucleotide‐activated‐*glycero‐manno*‐heptoses and an attractive, yet underexploited, target for development of broad‐spectrum antibiotics. We demonstrated that GmhA homologs in *Neisseria gonorrhoeae* and *N. meningitidis* (hereafter called GmhA_GC_ and GmhA_NM_, respectively) were interchangeable proteins essential for lipooligosaccharide (LOS) synthesis, and their depletion had adverse effects on neisserial viability. In contrast, the *Escherichia coli* ortholog failed to complement GmhA_GC_ depletion. Furthermore, we showed that GmhA_GC_ is a cytoplasmic enzyme with induced expression at mid‐logarithmic phase, upon iron deprivation and anaerobiosis, and conserved in contemporary gonococcal clinical isolates including the 2016 WHO reference strains. The untagged GmhA_GC_ crystallized as a tetramer in the closed conformation with four zinc ions in the active site, supporting that this is most likely the catalytically active conformation of the enzyme. Finally, site‐directed mutagenesis studies showed that the active site residues E65 and H183 were important for LOS synthesis but not for GmhA_GC_ function in bacterial viability. Our studies bring insights into the importance and mechanism of action of GmhA and may ultimately facilitate targeting the enzyme with small molecule inhibitors.

## 1 Introduction

The World Health Organization (WHO) and the Centers for Disease Control and Prevention (CDC) emphasized an urgent need for the development of antimicrobials with novel modes of action against antibiotic‐resistant threats with severe consequences for public health, including infections caused by drug‐resistant *Neisseria gonorrhoeae* (Centers for Disease Control and Prevention, 2013a, 2013b; World Health Organization, 2012, 2015). Gonorrhea is highly prevalent throughout the world, and if untreated or inadequately treated, often leads to serious repercussions on reproductive health including ectopic pregnancy, pelvic inflammatory disease, and infertility (Centers For Disease Control And Prevention, 2013b, Low, Unemo, Skov Jensen, Breuer, & Stephenson, 2014; World Health Organization, 2011). In the absence of a protective gonorrhea vaccine, antibiotics remain the sole therapeutic intervention. However, the well‐documented ability of gonococci to acquire antibiotic resistance continues to threaten available treatment options (Unemo, 2015; Unemo & Shafer, 2014). To meet the needs raised by WHO and CDC, our laboratory focuses on identification and validation of new molecular targets for the development of gonorrhea treatments (Bonventre, Zielke, Korotkov, & Sikora, 2016; Zielke, Wierzbicki, Baarda, & Sikora, 2015; Zielke, Wierzbicki, Weber, Gafken, & Sikora, 2014; Zielke et al., 2016).

Targeting the first enzymes in the nucleotide‐activated‐*glycero‐manno*‐heptose pathways is a relatively unexplored field, although it appears to be an alternative approach to the discovery of broad‐spectrum antibacterial drugs (Bauer, Stevens, & Hansen, 1998; Darby, Ananth, Tan, & Hinnebusch, 2005; Tamaki, Sato, & Matsuhashi, 1971; Valvano, Messner, & Kosma, 2002). Nucleotide‐activated‐*glycero‐manno*‐heptoses, while absent in eukaryotic cells, are widely present in bacteria and are crucial components of the lipopolysaccharides (LPS), lipooligosaccharides (LOS), capsules, O‐antigens, and glycan moieties of bacterial cell surface (S‐layer) glycoproteins (Valvano et al., 2002). In particular, one such potential drug target of significant interest is sedoheptulose‐7‐phosphate isomerase, GmhA, annotated previously as TfrA (Havekes, Lugtenberg, & Hoekstra, 1976) and LpcA (Brooke & Valvano, 1996a). GmhA is conserved in many Gram‐negative and some Gram‐positive bacteria and is responsible for catalyzing isomerization of the D‐sedoheptulose 7‐phosphate into D‐*glycero*‐α,β‐D‐*manno*‐heptose‐7‐phosphate (Eidels & Osborn, 1974), which is the first and common step for parallel biosynthetic pathways leading to generation of GDP‐D‐glycero‐α‐D‐*manno*‐heptose (D,D‐heptose) and ADP‐L‐glycero‐β‐D‐*manno*‐heptose [L,D‐heptose; reviewed in Ref: (Valvano et al., 2002)]. D,D‐heptose is required for glycosylation of flagella and capsular polysaccharides, and for the assembly of disaccharide repeating units (D,D‐heptose linked to L‐rhamnose) composing the S‐layer glycan in the Gram‐positive Eubacterium, *Aneurinibacillus thermoaerophilus* DSM 10155 (Eidels & Osborn, 1974; Kosma, Wugeditsch, Christian, Zayni, & Messner, 1995; Wugeditsch et al., 1999). L,D‐heptose is used for glycosylation of capsular polysaccharides (Valvano et al., 2002) and as a primary building block of LPS/LOS core oligosaccharide (Brooke & Valvano, 1996a; Eidels & Osborn, 1971). In addition, a large family of bacterial autotransporter heptosyltransferases (BAHTs) utilizes L,D‐heptose as a sugar donor to modify serine residues on their substrate autotransporters, which has a significant impact on the virulence of Gram‐negative pathogens (Lu, Li, & Shao, 2015). The L,D‐heptose is synthesized in sequential reactions catalyzed in order by GmhA‐HldE(HldA)‐GmhB‐HldE(HldC)‐HldD [reviewed in Refs: (Raetz & Whitfield, 2002; Valvano et al., 2002)]. Usually one or more L,D‐heptose molecules and two 2‐keto‐3‐deoxy‐D‐*manno*‐oct‐2‐ulosonic acid (KDO) residues form the inner portion (lipid A proximal) of the LPS/LOS core oligosaccharide, which is typically more conserved than the structurally diverse outer core (Raetz & Whitfield, 2002). Similarly, gonococcal LOS contain two basal heptose molecules, designated Hep I and Hep II, forming elongation centers α, β, and χ (Gibson et al., 1989; John, Griffiss, Apicella, Mandrell, & Gibson, 1991; Yamasaki, Bacon, Nasholds, Schneider, & Griffiss, 1991). The individual chains can be decorated with structures that mimic human carbohydrate epitopes (α chain linked to Hep I; Apicella & Mandrell, 1989); a single glucose, lactose, or glucose with additional sugars (β chain extending from Hep II; Gibson et al., 1989; Yamasaki et al., 1994); and GlcNac, GlcNac acetate, or occasionally galactose (χ chain; Mcleod Griffiss, Brandt, Saunders, & Zollinger, 2000). In addition, phosphate or phosphoethanolamine groups may be attached to the heptose residues (Preston, Mandrell, Gibson, & Apicella, 1996; Raetz & Whitfield, 2002). The phosphoric residues participate in the ionic interactions between LPS/LOS and outer membrane proteins, as well as divalent cations, providing a barrier against entry of detergents, antibiotics, and hydrophobic compounds (Preston et al., 1996; Raetz & Whitfield, 2002). The *N. gonorrhoeae* heptose‐monophosphate was recently linked with the clinical and epidemiological synergy of gonorrhea and HIV (Malott et al., 2013). At the molecular level, this interplay involves the unique ability of gonococci to efficiently liberate phosphorylated L,D‐heptose into the extracellular milieu, which elicits an immune response and induces HIV‐1 expression and viral production in cluster of differentiation 4‐positive (CD4^+^) T cells (Malott et al., 2013).

Mutations in genes encoding GmhA in different bacterial species examined to date resulted in pleiotropic effects including production of truncated LPS/LOS composed of lipid A and KDO residues, increased susceptibility to antibiotics and detergents, impaired biofilm formation, and attenuated virulence (Aballay, Drenkard, Hilbun, & Ausubel, 2003; Bauer et al., 1998; Brooke & Valvano, 1996b; Darby et al., 2005). In addition, lack of HldA, which acts immediately downstream from GmhA in the L,D‐heptose biosynthetic pathway, rendered gonococci unable to induce HIV‐1 expression (Malott et al., 2013). Therefore, we propose GmhA in *N. gonorrhoeae,* GmhA_GC_, as a molecular target for the development of new antigonococcal drugs. Herein, we performed characterization of GmhA_GC_ at the molecular, functional, and structural levels to facilitate the future targeting of this enzyme with small molecule inhibitors.

## 2 Experimental Procedures

### 2.1 Bacterial strains and growth conditions

Strains of bacteria used in this study are listed in Table 1. *Neisseria gonorrhoeae* and *N. meningitidis* were cultured either on gonococcal base solid medium (GCB, Difco), or in gonococcal base liquid (GCBL) medium supplemented with Kellogg's supplement I and II in ratios 1:100 and 1:1,000, respectively (Spence, Wright, & Clark, 2008). GCBL was additionally supplemented with sodium bicarbonate at a final concentration of 0.042%. *In vitro* host‐relevant growth conditions (iron deprivation, presence of normal human serum, anoxia) were procured as described previously (Zielke et al., 2016). *Neisseria* were cultured on solid media for 18–22 hr at 37°C in the presence of 5% atmospheric CO_2_. For *N. gonorrhoeae*, piliated or nonpiliated variants were passaged onto GCB and incubated for additional 18–22 hr. Colonies with piliated morphology were used for DNA transformation, while nonpiliated variants were used in all other experiments. *Escherichia coli* strains were grown either on Luria–Bertani agar (LBA, Difco) or cultured in Luria–Bertani broth (LB, Difco) at 37°C.

**Table 1:** Bacterial strains used in this study

Bacterial strains	Reference
*N. gonorrhoeae*	
FA1090	(Connell et al., 1988)
MS11	(Meyer, Mlawer, & So, 1982)
1291	(Apicella, Breen, & Gagliardi, 1978)
F62	(Sparling, 1966)
FA1090 ∆*gmhA*_*GC*_/*P*_*lac*_ *:: gmhA*_*GC*_	This study
FA1090 ∆*gmhA*_*GC*_/*P*_*lac*_ *:: gmhA*_*GC*_ E65A	This study
FA1090 ∆*gmhA*_*GC*_/*P*_*lac*_ *:: gmhA*_*GC*_ H183A	This study
FA1090 ∆*gmhA*_*GC*_/*P*_*lac*_ *:: gmhA*_*NM*_	This study
Baltimore collection 1991–1994:LGB1, LG14, LG2, LGB26, LG20	(Garvin et al., 2008; Zielke et al., 2014)
Clinical isolates from Public Health–Seattle & King County Sexually Transmitted Disease Clinic:UW01, UW02, UW03, UW04, UW05, UW06, UW07, UW08, UW09, UW10, UW11, UW12, UW13	(Zielke et al., 2016)
2016 WHO reference strains:F, G, K, L, M, N, O, P, W, X, Y, Z, U, V	(Unemo et al., 2016)
*N. meningitidis*	
MC58	(McGuinness et al., 1991)
MC58 ∆*gmhA*_*NM*_/*P*_*lac*_ *:: gmhA*_*NM*_	This study
MC58 ∆*gmhA*_*NM*_/*P*_*lac*_ *:: gmhA*_*GC*_	This study
*E. coli*	
MC1061	(Casadaban & Cohen, 1980)
BL21(DE3)	(Studier & Moffatt, 1986)

Antibiotics were used on selected bacteria in the following concentrations: for *N. gonorrhoeae*: kanamycin 40 μg/ml, erythromycin 0.5 μg/ml; for *N. meningitidis*: kanamycin 80 μg/ml, erythromycin 2 μg/ml; for *E. coli*: kanamycin 50 μg/ml, erythromycin 250 μg/ml.

### 2.2 Genetic manipulations, site‐directed mutagenesis, and transcomplementation

Plasmids and primers used in this study are listed in Table S1. Oligonucleotides were designed based on the genomic sequence of *N. gonorrhoeae* FA1090 (NC_002946), *N. meningitidis* MC58 (NC_003112), and *E. coli* BL21(DE3) (NC_012892) using SnapGene software version 2.8 (GSL Biotech LLC) and synthesized by IDT Technologies. Genomic DNA of gonococcal strains, *N. meningitidis* MC58, and *E. coli* BL21(DE3) was isolated with the Wizard Genomic DNA Purification Kit (Promega). PCR products and plasmid DNA were purified using QIAprep Spin Miniprep Kit (QIAGEN). PCR reactions were performed using chromosomal or plasmid DNA as template, appropriate oligonucleotides, and Q5^®^ High‐Fidelity DNA Polymerase (NEB). *E. coli* MC1061 was used as the host during the molecular cloning and site‐directed mutagenesis procedures. All created constructs and suppressor mutations in ∆*gmhA*_*GC*_/*P*_*lac*_ *::gmhA*_*GC*_ were verified by Sanger Sequencing at the Center for Genomic Research and Biocomputing at Oregon State University. Transformation of *N. gonorrhoeae* and *N. meningitidis* was performed as described previously (Alexander, Richardson, & Stojiljkovic, 2004; Zielke et al., 2014).

The recombinant GmhA_GC_ (rGmhA_GC_) containing N‐terminal‐6 × His‐tag followed by the tobacco etch virus (TEV) protease recognition site was obtained by amplifying the DNA region of the *ngo1986* lacking stop codon with primers rNGO1986‐F and rNGO1986‐R and cloning the obtained PCR product (615 bp) into NcoI/HindIII sites of pRSF‐NT (Table S1).

The conditional GmhA_GC_ mutant, FA1090 ∆*gmhA*_*GC*_/*P*_*lac*_ *::gmhA*_*GC*_, was constructed using a strategy as described by (Zielke et al., 2016) by placing an additional copy of *ngo1986* under the control of the isopropyl β‐D‐1‐thiogalactopyranoside (IPTG)‐inducible promoter, P_*lac*_, within an intergenic region located between *lctP* and *aspC* in the FA1090 chromosome (Mehr, Long, Serkin, & Seifert, 2000) and a subsequent in‐frame replacement of the *ngo1986* in its native chromosomal locus with the nonpolar kanamycin resistance cassette. Specifically, the *ngo1986* containing its native ribosomal binding site (RBS) was amplified with primers NGO1986‐RBS‐F and NGO1986‐RBS‐R. The resulting 628 bp PCR product was digested with FseI and inserted into ScaI/FseI‐treated pGCC4, yielding pGCC4‐GmhA_GC_. After transformation of FA1090 with pGCC4‐GmhA_GC_, gonococci were selected on GCB with 0.5 μg/ml erythromycin and verified by PCR with primers pGCC4‐Ver‐F and pGCC4‐Ver‐R.

The constructs for deletion of *gmhA*_*GC*_ were obtained by amplification of the 552 bp upstream DNA region and 571 bp downstream from *ngo1986* with primers NGO1986‐UP‐F/NGO1986‐UP‐R and NGO1986‐Down‐F/NGO1986‐Down‐R, respectively. The upstream fragment was digested with EcoRI/KpnI and cloned into similarly cleaved pUC18K, yielding pUC18K‐GmhA_GC_‐Up. Next, the downstream fragment was inserted into the BamHI/HindIII‐cleaved pUC18K‐GmhA_GC_‐Up. The resulting pUC18K‐GmhA_GC_ was linearized with HindIII and used to transform FA1090 carrying the second copy of GmhA on the chromosome, FA1090*P*_*lac*_ *::gmhA*_*GC*_. Clones were selected on a solid medium supplemented with kanamycin and 0.1 mmol/L IPTG, and verified by PCR with NGO1986‐Ver‐F and NGO1986‐Ver‐R primers. Subsequently, selected gonococci were further verified by immunoblotting analyses using anti‐GmhA_GC_ antisera.

To generate GmhA_GC_E65A and GmhA_GC_H183A, the template for site‐directed mutagenesis (pUC18‐GmhA_GC_) was constructed by amplifying *ngo1986* with its native RBS using primers NGO1986‐RBS‐F and NGO1986‐RBS‐R and cloning into SmaI‐cleaved pUC18. Site‐directed mutagenesis was performed using the template; primer pairs E65A‐F/E65A‐R or H183A‐F/H183A‐R, respectively, and Q5 Site‐Directed Mutagenesis Kit (NEB), according to the manufacturer's manual. Subsequently, the mutated variants of GmhA_GC_ were amplified with NGO1986‐RBS‐F and NGO1986‐RBS‐R, cleaved with FseI, cloned into ScaI/FseI‐digested pGCC4, introduced into the FA1090 chromosome, and the native *ngo1986* was deleted as described above.

For transcomplementation studies, GmhA homologs from *N. meningitidis* MC58 (*nmb2090*) and *E. coli* BL21(DE3) (*ECBD_3400*) were amplified with primer pairs NGO1986‐RBS‐F/NGO1986‐RBS‐R and ECBD3400‐RBS‐F, respectively. The resulting PCR products were digested with FseI and introduced into ScaI/FseI‐treated pGCC4. Introduction into the FA1090 chromosome and deletion of GmhA were performed as outlined above.

The cytoplasmic marker control for subfractionation experiments, the *zwf* gene (*ngo0715*) encoding glucose‐6‐phosphate‐1‐dehydrogenase was amplified using primers RAZ548 and RAZ549. The PCR product was cleaved with NcoI and HindIII and cloned into similarly digested pRSF‐NT to obtain plasmid pRSF‐NT‐Zwf.

### 2.3 Proteome subfractionation procedures

Colonies of *N. gonorrhoeae* FA1090 were collected from GCB, suspended in 500 ml of GCBL to OD_600_ of 0.1, and cultured with aeration at 37°C until OD_600_ of ~0.8. Bacterial cells were separated from the suspension by centrifugation (10 min, 6,000*g*, 4°C). The crude cell envelopes were purified using a sodium carbonate extraction procedure while naturally released membrane vesicles (MVs) and soluble proteins (SS) were fractionated from culture supernatants by ultracentrifugation as described previously (Zielke et al., 2016).

### 2.4 GmhA_GC_ depletion studies

Colonies of FA1090 ∆*gmhA*_*GC*_/*P*_*lac*_ *:: gmhA*_*GC*_ were collected with a cotton swab from GCB supplemented with 10 μmol/L IPTG and suspended in GCBL to OD_600_ of 0.1. After two washes in prewarmed GCBL, bacterial suspension was split and incubated with shaking (220 rpm) with or without IPTG for 3 hr at 37°C. Cultures were back‐diluted to OD_600_ of 0.1 in fresh GCBL, as described above, and cultured for additional 6 hr. At specific time points indicated in the text, OD_600_ measurements were taken, samples for western blotting and LOS isolation were withdrawn, and cultures were serially diluted followed by plating on GCB for enumeration of colony‐forming units (CFUs). Experiments were performed on three separate occasions and mean values and SEM are presented.

### 2.5 Isolation of LOS and silver staining

LOS was isolated from *N. gonorrhoeae* and *N. meningitidis* based on the method described previously (Hitchcock & Brown, 1983). Bacteria were either collected from GCB or GCBL, as specified in the text, suspended in 1.5 ml of GCBL to OD_600_ of 0.2 and spun down for 1.5 min at 15,000*g*. Pelleted cells were lysed by addition of 50 μl of lysis buffer (2% SDS, 4% β‐mercaptoethanol, 10% glycerol, 1mol/L Tris‐HCl pH 6.8, and 0.01% bromophenol blue) and incubation at 100°C for 10 min. Samples were allowed to cool down to room temperature and proteins were digested by addition of 25 μg proteinase K in 10 μl of lysis buffer and incubated for 1 hr at 60°C. Isolated LOS was resolved on 16.5% Mini‐PROTEAN^®^ Tris‐Tricine Gel (Bio‐Rad) and visualized by a silver staining procedure (Tsai & Frasch, 1982).

### 2.6 Fitness assessment

Colonies of different neisserial strains, as indicated in the text, were collected from GCB and reconstituted in GCBL to OD_600_ of 0.1. Bacterial cultures were incubated in the absence of IPTG for 3 hr at 37°C with aeration and subsequently back‐diluted to OD_600_ of 0.2, serially diluted, and plated on GCB with or without IPTG for CFUs scoring. Experiments were repeated in three biological replicates and mean values with corresponding SEM are presented.

### 2.7 Purification of the rGmhA_GC_ and rZwf and production of polyclonal rabbit antibodies

*E. coli* BL21(DE3) strain carrying either pRSF‐NT‐GmhA_GC_ or pRSF‐NT‐Zwf was used as heterologous host for overproduction and purification of rGmhA_GC_ and recombinant Zwf (rZwf), respectively. Overnight cultures were back‐diluted in 2.0 L of LB supplemented with kanamycin and incubated with aeration at 37°C until the optical density (OD_600_) reached ~0.5. Overproduction of rGmhA_GC_ and rZwf was induced with 0.1 and 1 mmol/L IPTG, respectively, and cultures were incubated for additional 3 hr at 37°C. Cells were harvested by centrifugation (6,000*g*, 10 min, 4°C). Pelleted bacteria carrying pRSF‐NT‐GmhA_GC_ were suspended in lysis buffer (20 mmol/L Tris‐HCl pH 7.0, 1 mol/L NaCl, 10 mmol/L imidazole, 5% glycerol) supplemented with a Pierce Protease Inhibitor Mini Tablet (Thermo Scientific) and lysed by passaging six times through a French pressure cell at 12,000 psi. Cell debris and unbroken cells were separated from soluble protein fraction by centrifugation at 16,000*g* for 30 min at 4°C. The supernatant was passed through 0.22 μm membrane filter (VWR International) and applied onto Bio‐Scale MiniProfinity IMAC cartridges (Bio‐Rad) on the NGC Scout Chromatography system (Bio‐Rad). Loosely bound proteins were removed with 10 column volumes of wash buffer (20 mmol/L Tris pH 8.0, 500 mmol/L NaCl, 40 mmol/L imidazole) and elutions were conducted with a 40–250 mmol/L imidazole gradient. Fractions containing rGmhA_GC_ were combined and a PD‐10 column (GE Healthcare) was used to exchange the buffer to 20 mmol/L HEPES pH 7.5, 100 mmol/L NaCl, 5% glycerol. Subsequently, the N‐terminal‐6 × His‐tag of the rGmhA_GC_ was removed by TEV protease. Cleavage reaction was prepared by mixing rGmhA_GC_ with TEV in 20:1 (w:w ratio) in 500 μl of cleavage buffer (0.5 mol/L Tris‐HCl pH 8.0, 5 mmol/L EDTA), 10 μl of 0.5 mol/L DTT, and 500 μl of Ni‐NTA agarose (Qiagen) equilibrated with cleavage buffer. Following overnight incubation at room temperature, cleavage mixture was loaded onto a 5 ml polypropylene column (Thermo Scientific) and supernatant containing 6 × His‐tag‐free rGmhA_GC_ was collected. To remove residual TEV protease that coeluted with rGmhA_GC_, the mixture was incubated again with 500 μl of Ni‐NTA agarose (Qiagen) equilibrated with cleavage buffer for 1 hr with rotation at 4°C, and rGmhA_GC_ was eluted. The purified rGmhA_GC_ was applied to a PD‐10 column (GE Healthcare) for a buffer exchange into 20 mmol/L HEPES pH 7.5, 100 mmol/L NaCl, and 5% glycerol.

Purification of rZwf was accomplished using the same procedures as described above with the following modifications. *E. coli* cells were resuspended in lysis buffer (20 mmol/L Tris‐HCl pH 8.0, 10 mmol/L imidazole, 450 mmol/L NaCl). Cells were lysed using French Press and rZwf was purified using 5 ml Bio‐Scale Mini Nuvia IMAC Ni‐Charged column (Bio‐Rad) connected to a NGC Chromatography System (Bio‐Rad). Bound peptides were eluted using elution buffer (20 mmol/L Tris‐HCl pH 8.0, 250 mmol/L imidazole, 450 mmol/L NaCl). Fractions containing proteins were pooled, EDTA and DTT were added to final concentrations of 0.5 mmol/L and 1 mmol/L, respectively, and the His‐tag was removed by overnight incubation with TEV protease (ratio 1:100 w/w). After cleavage, the proteins were separated using Hi Load 16/600 Superdex 75 pg column (GE Healthcare) and buffer containing 20 mmol/L Tris pH 8, 150 mmol/L NaCl connected to the NGC Chromatography System. Fractions containing Zwf were pooled together and concentrated using 10 kDa Vivaspin 20 concentrators (GE Healthcare). Glycerol was added to a final concentration of 10% and the protein was aliquoted and stored at −80°C.

The polyclonal rabbit anti‐GmhA_GC_ and anti‐Zwf antibodies were generated using 6 × His‐tag‐free rGmhA_GC_ and rZwf, respectively. Standard 13‐week antibody production protocols were applied, utilizing four New Zealand White rabbits, animal handling was performed according to the Animal Protocol #1 approved by IACUC, in a certified animal facility (USDA 93‐R‐283) and the NIH Animal Welfare Assurance Program (#A4182‐01) at the Pacific Immunology Corporation. Anti‐GmhA_GC_ and anti‐Zwf antisera were used at 1:10,000.

### 2.8 Size exclusion chromatography

The NGC Scout Chromatography system (Bio‐Rad) employing a HiLoad 16/600 Superdex 75 pg column (GE Healthcare Life Sciences) was used to separate purified rGmhA_GC_ based on the molecular size. Buffer for the chromatography (20 mmol/L Tris‐HCl pH 8.0, 500 mmol/L NaCl) was applied at 1 ml/min flow rate. Gel Filtration Standard (Bio‐Rad) was used to determine the size of the separated proteins.

### 2.9 Crystallization and structure determination of *N. gonorrhoeae* GmhA_GC_

The screening for initial crystallization conditions was performed using JCSG Core Suites I‐IV (Qiagen) (Newman et al., 2005). The optimized crystals were grown using 0.1 mol/L Tris‐HCl pH 8.5, 0.2 mol/L magnesium chloride, 30% PEG4000. Crystals were transferred to a cryoprotectant solution supplemented with 20% glycerol and flash‐frozen in liquid nitrogen. The diffraction data were collected from a single crystal at the beamline 22‐ID, Southeast Regional Collaborative Access Team (SER‐CAT) at the Advanced Photon Source, Argonne National Laboratory. Data were integrated and scaled using *XDS* and *XSCALE* (Kabsch, 2010a). The structure was solved by molecular replacement using Phaser (McCoy et al., 2007) and structure of *Pseudomonas aeruginosa* GmhA (PDB 3BJZ) as a search model (Taylor et al., 2008). The electron density modification was performed using Parrot (Cowtan, 2010) followed by automated model rebuilding using Buccaneer (Cowtan, 2006). The model was completed by manual rebuilding in *Coot* (Emsley, Lohkamp, Scott, & Cowtan, 2010) and was refined using REFMAC5 (Murshudov et al., 2011). The structure was validated using *Coot* and the MolProbity server (Chen et al., 2010). The structural figures were generated using PyMol (The PyMOL Molecular Graphics System, Version 1.8 Schrödinger, LLC.).

### 2.10 SDS‐PAGE and immunoblotting

Whole cell lysates of *Neisseria* strains indicated in the text were either collected from solid media after 22 hr of aerobic or 48 hr of anaerobic growth, or harvested from liquid media following procedures described (Zielke et al., 2014, 2016). *E. coli* cells were collected from LB agar plates after overnight incubation. Samples were normalized based on OD_600_ values or based on total protein concentration, and SDS‐PAGE, staining with Coomassie brilliant blue G‐250, and immunoblotting analyses were performed exactly as described previously (Zielke et al., 2014, 2016).

### 2.11 Statistical analysis

GraphPad Prism's build‐in *t*‐test was used for determination of statistically significant differences between obtained experimental results. A confidence level of 95% was used for all analyses.

### 2.12 Accession numbers

The coordinates and structure factors were deposited to the Protein Data Bank with accession code 5I01.

## 3 Results

### 3.1 Chromosomal location and purification of GmhA_GC_

Genes encoding the enzymes of L,D‐heptose biosynthesis pathway are scattered throughout the chromosome in the majority of bacteria including *N. gonorrhoeae* (Valvano et al., 2002). The GmhA homolog in *N. gonorrhoeae* strain FA1090 is encoded by *ngo1986*, which is located between *ngo1985*, coding for an outer membrane lipoprotein (Zielke et al., 2014) and *ngo1987* encoding a putative endonuclease (YraN). Comparison of the *gmhA* location using BioCyc Pathway/Genome Database Collection (http://biocyc.org/) showed that this genetic arrangement is conserved among the various deposited *Neisseria* species and isolates (*n *=* *70) with only a few exceptions, including *N. bacilliformis* ATCC BAA‐1200, *N. sp*. oral taxon 020 F0370, *N. shayeganii* 871, and *N. subflava* NJ9703.

GmhA_GC_ consists of 197 amino acids with residues 37–197 comprising a sugar isomerase domain (SIS) (Bateman, 1999) that is shared between all ketose/aldose isomerases (Golinelli‐Pimpaneau, Le Goffic, & Badet, 1989). At the amino acid level, GmhA_GC_ shows 43–57% identity with crystallized orthologous proteins (Table 3) and contains the key conserved residues observed in all GmhA homologs, including three serine residues and a threonine that presumably interact with the phosphate group of sedoheptulose 7‐phosphate or D‐*glycero*‐D‐*manno*‐heptose 7‐phosphate (Harmer, 2010; Valvano et al., 2002).

To characterize GmhA_GC_, we first purified recombinant protein with an N‐terminal 6 × His‐tag followed by the tobacco etch virus (TEV) protease cleavage site and prepared untagged protein. Size exclusion chromatography indicated that the native GmhA_GC_ forms tetrameric structures in the solution (Fig. S1A). The denatured protein migrated on SDS‐PAGE according to the predicted molecular mass of 21.093 kDa (Fig. S1B). Untagged GmhA_GC_ was subsequently used in crystallization and to obtain polyclonal rabbit antisera.

### 3.2 *N. gonorrhoeae* deprived of GmhA_GC_ displays defect in LOS synthesis and growth cessation

**Figure 1:**
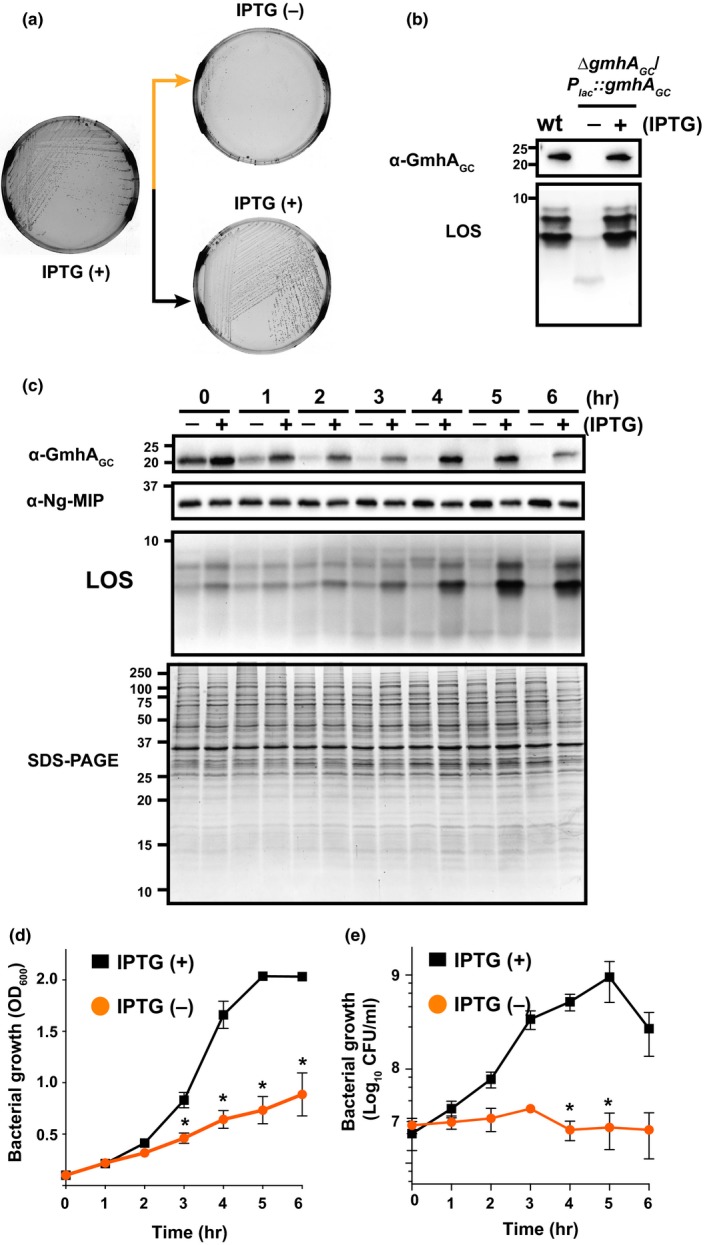
GmhA_GC_ is pivotal for *N. gonorrhoeae* growth and lipooligosaccharide (LOS) synthesis. (a) The *N. gonorrhoeae* FA1090 *gmh* *A*_*GC*_ conditional knockout strain, ∆*gmh* *A*_*GC*_/*P*_*lac*_ *::gmh* *A*_*GC*_, was streaked out from frozen glycerol stock on solid media supplemented with 20 μmol/L isopropyl β‐D‐1‐thiogalactopyranoside (IPTG). After 18 hr incubation at 37°C in the presence of 5% atmospheric CO_2_, the colonies were passaged onto plates either with (+) or without IPTG (−). (b) Whole cell lysates of FA1090 wild‐type and isogenic ∆*gmh* *A*_*GC*_/*P*_*lac*_ *::gmh* *A*_*GC*_ harvested from plates with (+) or without 20 μmol/L IPTG (−) were either probed with polyclonal rabbit antisera or subjected to LOS extraction using proteinase K followed by silver staining. (c–e) The FA1090 ∆*gmh* *A*_*GC*_/*P*_*lac*_ *::gmh* *A*_*GC*_ cells were collected from solid media supplemented with 20 μmol/L IPTG, suspended to OD_600_ of 0.1, washed twice, divided, and cultured either in the presence or absence of IPTG for 3 hr. At time point designated as 0 hr, corresponding cultures were back‐diluted to the same density (OD_600_ of 0.1) into fresh media with or without IPTG and incubated for additional 6 hr. Samples of bacterial cultures were collected every hour for GmhA_GC_ and Ng‐MIP immunoblotting analysis, LOS, and whole cell protein profiles (c), monitoring of bacterial proliferation by measurement of density of the cultures at OD_600_, (d) and spotting serially diluted bacteria on solid media with IPTG for CFU scoring (e). Whole cell lysates were matched by the same OD_600_ units. As loading control, samples separated by SDS‐PAGE were either probed with anti‐Ng‐MIP antibodies or stained with Coomassie brilliant blue G‐250. The migration of molecular mass marker (kDa) is indicated on the left. All experiments were performed in three biological replicates. Means and SEM are presented on graphs; *p <* *.05

The genetic inactivation of *gmhA* in *E. coli*,* Haemophilus influenzae*,* H. ducreyi*,* Salmonella enterica*, and *Yersinia pestis* resulted in altered outer membrane permeability and decreased virulence in animal infection models, however, the mutants did not display drastic growth defects *in vitro* (Aballay et al., 2003; Bauer et al., 1998; Brooke & Valvano, 1996b; Darby et al., 2005). Therefore, we were surprised by the failure of our numerous attempts to generate a clean deletion of *ngo1986* in *N. gonorrhoeae* FA1090. As an alternative strategy, an additional copy of *gmhA*_GC_ was placed under the control of the IPTG‐inducible *P*_*lac*_ promoter and introduced into the chromosome of wild‐type FA1090, followed by an allelic replacement of *ngo1986* with the kanamycin resistance cassette. The obtained ∆*gmhA*_*GC*_/*P*_*lac*_ *::gmhA*_*GC*_ mutant grew scarcely after passage on solid media without IPTG, whereas abundant colonies were observed in the presence of the inducer (Figure 1a). The depletion of GmhA_GC_ was corroborated by probing whole cell lysates of these gonococci with anti‐GmhA_GC_ antisera (Figure 1b). Visualization of LOS species by Tricine‐SDS‐PAGE coupled with silver staining showed, as expected, that bacteria deprived of GmhA_GC_ carried a truncated version of LOS that migrated faster in comparison with the LOS species isolated from wild‐type and ∆*gmhA*_*GC*_/*P*_*lac*_ *::gmhA*_*GC*_ grown in the presence of IPTG (Figure 1b).

**Figure 2:**
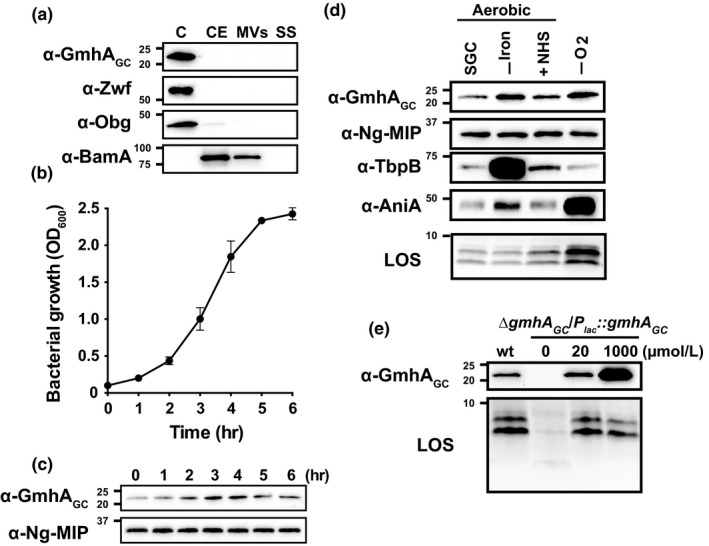
Assessment of GmhA_GC_ subcellular localization and expression patterns. (a) *N. gonorrhoeae* wild‐type FA1090 cells were harvested during mid‐exponential phase and subjected to proteome extraction to separate cytoplasmic/periplasmic proteins (C), cell envelopes (CE), naturally released membrane vesicles (MVs), and soluble proteins in culture supernatants (SS). Individual fractions were loaded based on the total amount of protein (μg) and probed with antisera as indicated on the left. (b–c) Wild‐type *N. gonorrhoeae* FA1090 was cultured in liquid media and at indicated time points, OD_600_ measurements were taken (b) and samples were withdrawn and processed for immunoblotting analysis of GmhA_GC_ and Ng‐MIP (c). (d) Quantities of GmhA_GC_ and lipooligosaccharide (LOS) in wild‐type FA1090 during *in vitro* conditions relevant to different infection sites [standard growth under aerobic conditions (SGC), iron deprivation (‐Iron), presence of normal human serum (+NHS), and anaerobiosis (‐O_2_)], were assessed by probing the whole cell lysates with anti‐GmhA_GC_ antibodies and silver staining of proteinase K‐extracted LOS, respectively. Immunoblotting experiments with antisera against Ng‐MIP, TbpB, and AniA were used as markers for ubiquitous expression, iron‐limiting, and anaerobic conditions, respectively. (e**)** Effect of overexpression of GmhA_GC_ on LOS amounts was examined by harvesting wild‐type FA1090 and isogenic ∆*gmh* *A*_*GC*_/*P*_*lac*_ *::gmh* *A*_*GC*_ grown on solid media with different isopropyl β‐D‐1‐thiogalactopyranoside (IPTG) concentrations (0, 20, or 1,000 μmol/L). Whole cell lysates were either probed with anti‐GmhA_GC_ antisera or treated with proteinase K and LOS was visualized by silver staining. Experiments were performed in biological triplicates and representative immunoblots and silver‐stained gels are shown. Mean values and corresponding SEM are presented on the graph. Migration of a molecular mass marker (kDa) is indicated on the left

Subsequently, time course experiments under standard laboratory growth conditions were conducted to examine the correlation between depletion of GmhA_GC_, LOS formation, and *N. gonorrhoeae* fitness. An initial suspension of nonpiliated colonies of ∆*gmhA*_*GC*_/*P*_*lac*_ *::gmhA*_*GC*_ collected from solid media supplemented with IPTG was divided and cultured in liquid media in the presence or absence of IPTG [(+) or (−), respectively]. After 3 hr, to ensure GmhA_GC_ depletion, the corresponding cultures were diluted to OD_600_ of 0.1 in media with or without IPTG. From this point onward (shown as 0 hr in Figure 1c–e), samples were withdrawn every hour to assess GmhA_GC_ levels and LOS formation (Figure 1c), culture density (Figure 1d), and colony forming units (CFUs; Figure 1e). Immunoblotting with anti‐GmhA_GC_ antisera showed that in the absence of IPTG, GmhA_GC_ levels were slightly decreased at 0 hr and complete GmhA_GC_ depletion was achieved three hours later (Figure 1c). Also, it was noted that despite the presence of inducer, GmhA_GC_ had a peculiar expression pattern; decreased amounts of protein in comparison with these observed at 0 hr were detected at 1 hr and in early‐log phase (corresponding to 2–3 hr data points). Furthermore, increased GmhA_GC_ levels were found in late‐log phase (4, 5 hr) followed by a decline in stationary phase (6 hr; Figure 1c–e). In contrast, no major differences were noticed in the overall protein profiles and the level of Ng‐MIP (Figure 1c), which is ubiquitously expressed throughout phases of gonococci growth (Zielke et al., 2016). As expected based on data presented in Figure 1b, the wild‐type LOS species diminished over time and were barely detectable upon GmhA_GC_ diminution (Figure 1c). Finally, analysis of culture density and number of viable gonococci showed that in media lacking IPTG, bacterial growth was greatly reduced concomitant to depletion of GmhA_GC_ (Figure 1c–e), whereas in the presence of IPTG, the generation time of the ∆*gmhA*_*GC*_/*P*_*lac*_ *::gmhA*_*GC*_ mutant was 61.2 min (Figure 1d–e), a value similar to the parental wild‐type strain (56.5 min; Figure 2b). In addition, under nonpermissive conditions, we observed that the OD_600_ of the mutant culture continued to increase (Figure 1d) while the number of viable bacteria remained at 10^7^ CFUs/ml (Figure 1e). These results suggested that suppressors were arising in the mutant population but had not reached the point where they overcome the persistent population by the end of the 6‐hr growth curve. We tested this possibility by passaging ∆*gmhA*_*GC*_/*P*_*lac*_ *::gmhA*_*GC*_ on solid media without and with IPTG. After the third passage, ample bacterial colonies were obtained with several mutations within the *P*_*lac*_ *,* allowing expression of GmhA_GC_ and LOS synthesis (Fig. S2).

Together, these findings demonstrated that GmhA_GC_ is pivotal for LOS synthesis and optimal growth of *N. gonorrhoeae*.

### 3.3 GmhA_GC_ subcellular localization and expression patterns

Preliminary results and absence of signal peptide suggested that GmhA of *E. coli* is a cytoplasmic enzyme (Brooke & Valvano, 1996a). However, subcellular localization of GmhA and expression patterns were never assessed. To examine the localization of GmhA_GC_, *N. gonorrhoeae* FA1090 was harvested at mid‐logarithmic phase of growth and subjected to subfractionation procedures. Equal amounts of extracted proteome fractions including cytoplasm, cell envelopes, naturally released membrane vesicles, and soluble proteins in culture supernatants were separated by SDS‐PAGE and probed with polyclonal antibodies (Figure 2a). GmhA_GC_ was found solely in the cytoplasmic protein fraction, similarly to the cytoplasmic enzyme glucose‐6‐phosphate 1‐dehydrogenase, Zwf. As expected, the GTPase Obg (Obg_GC_), which primarily associates with 50S ribosomal subunits and partly with the peripheral inner membrane proteome (Papanastasiou et al., 2013; Zielke et al., 2015), was detected mainly in the cytoplasm and minute amounts were also found in the cell envelope fraction; whereas, antisera against the outer membrane protein marker BamA (Zielke et al., 2016) cross‐reacted with fractions containing cell envelopes and membrane vesicles.

The expression of GmhA_GC_ was subsequently examined in wild‐type FA1090 during routine aerobic growth in liquid media (Figure 2b–c) and on solid media under conditions that more closely mimic clinical infection, such as iron deprivation, exposure to human serum, and anoxia (Figure 2d). Antibodies against Ng‐MIP, TbpB, and AniA were used as markers for ubiquitous expression, iron‐limiting conditions, and anaerobiosis, respectively (Cornelissen, 2008; Zielke et al., 2014, 2016). Immunoblotting analyses of whole cell lysates showed that expression of GmhA_GC_ peaked during mid‐exponential phase (3 and 4 hr, Figure 2b–c). Increased GmhA_GC_ levels were also detected during iron deprivation and anaerobic growth in comparison with standard laboratory conditions (Figure 2d). As expected, expression of Ng‐MIP remained constant throughout different phases of gonococcal growth and was unchanged under all tested conditions (Zielke et al., 2015, 2016), while TbpB and AniA were the most highly upregulated during iron deprivation and anaerobic growth, respectively (Figure 2d).

The increase in GmhA_GC_ expression did not influence LOS migration patterns in wild‐type gonococci, albeit increased amounts of total LOS were observed in bacteria cultured in the presence of normal human serum and anaerobically (Figure 2d). To further assess the possible correlation between expression of GmhA_GC_ and LOS levels, ∆*gmhA*_*GC*_/*P*_*lac*_ *::gmhA*_*GC*_ was cultured in increasing concentrations of IPTG. The vast overexpression of GmhA_GC_ achieved with 1,000 μmol/L IPTG did not have adverse effect on bacterial growth (data not shown) and had no effect on the LOS quantities (Figure 2e).

Cumulatively, these experiments demonstrated that GmhA_GC_ is a cytoplasmic enzyme with augmented expression during mid‐logarithmic phase, iron depletion and anaerobiosis, and that overproduction of GmhA_GC_ alone does not alter gonococcal LOS abundance.

### 3.4 Conservation of *gmhA* among *Neisseria*

Analysis of *gmhA* conservation showed that the gene (locus NGO1986, NMB2090, NMC2070) is present in all of the 39,182 *Neisseria spp*. genomes deposited into the PubMLST database (http://pubmlst.org/neisseria/ as of July, 20, 2016) and that there are 340 alleles and 323 single nucleotide polymorphic sites (Fig. S4).

**Figure 3:**
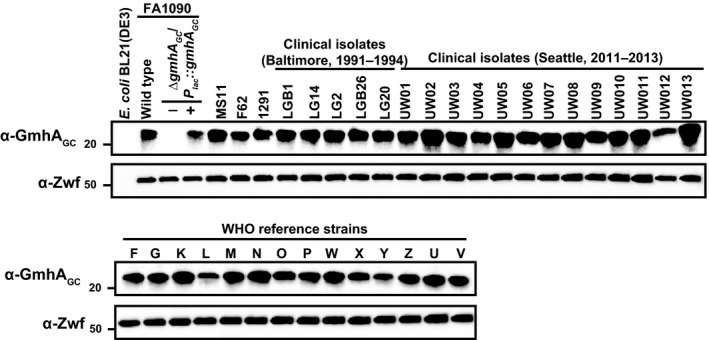
GmhA_GC_ expression in a panel of *N. gonorrhoeae* isolates. Whole cell lysates of *E. coli*, wild‐type FA1090, ∆*gmh* *A*_*GC*_/*P*_*lac*_ *::gmh* *A*_*GC*_ grown either with (+) or without (−) isopropyl β‐D‐1‐thiogalactopyranoside (IPTG), and 36 additional strains of *N. gonorrhoeae* were resolved on a 10–20% Tris‐Glycine gel and probed with anti‐GmhA_GC_ antibodies. Immunoblotting with anti‐Zwf antisera was used as a loading control. Migration of a molecular mass marker (kDa) is indicated on the left

Expression of GmhA_GC_ among 36 different *N. gonorrhoeae* strains isolated from patients at different times and geographic locations, including the 2016 WHO reference strains (Unemo et al., 2016), was also assessed by immunoblotting. Whole cell lysates were resolved by SDS‐PAGE and probed with either polyclonal anti‐GmhA_GC_ antisera or Zwf antibodies, or stained with Coomassie brilliant blue G‐250 as a loading control. Antisera against GmhA_GC_ cross‐reacted with all clinical isolates of *N. gonorrhoeae*, but no cross‐reactivity was detected for the *E. coli* GmhA homolog (Figure 3). In addition, there were noticeable differences in GmhA_GC_ protein abundance between the strains, while Zwf was uniformly expressed and there were no discrepancies in regard to samples normalization and loading (Figure 3 and Fig. S3).

### 3.5 Hindering GmhA_GC_ isomerase activity does not influence *N. gonorrhoeae* growth

**Figure 4:**
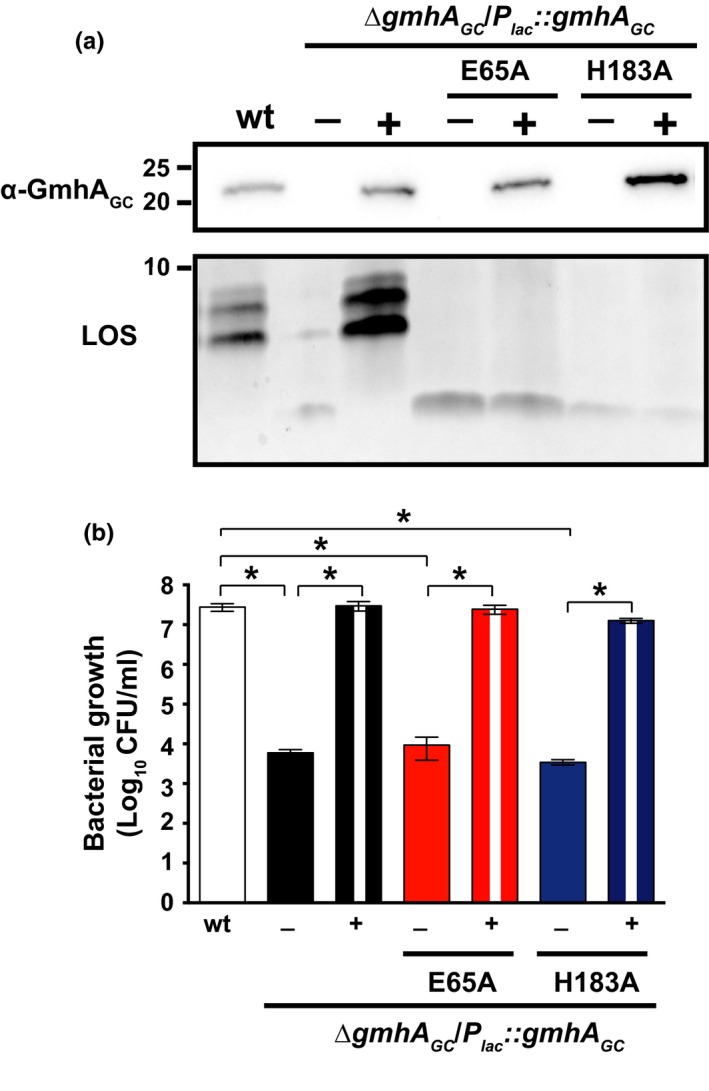
Hindering GmhA_GC_ isomerase activity does not influence *N. gonorrhoeae* growth. (a) Wild‐type FA1090 and isogenic conditional mutants carrying either native *gmh* *A*_*GC*_ (∆*gmh* *A*_*GC*_/*P*_*lac*_ *::gmh* *A*_*GC*_) or mutated variants of GmhA_GC_ (∆*gmh* *A*_*GC*_/*P*_*lac*_ *::gmh* *A*_*GC*_E65A or ∆*gmh* *A*_*GC*_/*P*_*lac*_ *::gmh* *A*_*GC*_H183A) were collected from solid media with (+) and without (−) isopropyl β‐D‐1‐thiogalactopyranoside (IPTG). Expression of individual GmhA_GC_ variants and lipooligosaccharide (LOS) patterns were examined in whole cell extracts by immunoblotting and silver staining, respectively. Samples were matched by equivalent OD_600_ units. Migration of a molecular mass marker (kDa) is indicated on the left. (b) Wild‐type FA1090 and conditional mutants ∆*gmh* *A*_*GC*_/*P*_*lac*_ *::gmh* *A*_*GC*_, ∆*gmh* *A*_*GC*_/*P*_*lac*_ *::gmh* *A*_*GC*_E65A, and ∆*gmh* *A*_*GC*_/*P*_*lac*_ *::gmh* *A*_*GC*_H183A were collected from solid media supplemented with 20 μmol/L IPTG, suspended in liquid media to OD_600_ of 0.1, cultured for 3 hr, back‐diluted to equal OD_600_ of 0.2, serially diluted, and spotted on solid media in the presence (+) and absence (−) of IPTG. CFUs were scored. The data show averages of CFUs with corresponding SEM of at least three separate experiments; *p <* *.05

The crucial side chains for GmhA enzymatic activity appeared to be E65 and H180 in the *E. coli* ortholog as analyzed by in vitro kinetic assays, LOS synthesis, and novobiocin sensitivity studies. It has been proposed that these two residues act as the base and the acid, respectively, to promote the isomerization reaction of D‐sedoheptulose 7‐phosphate into D‐*glycero*‐α,β‐D‐*manno*‐heptose‐7‐phosphate (Taylor et al., 2008). Therefore, to determine whether the observed decrease in *N. gonorrhoeae* survival upon GmhA_GC_ depletion is a consequence of abolished LOS synthesis, site‐directed mutagenesis of corresponding residues (E65 and H183 in GmhA_GC_) was employed. The obtained GmhA_GC_ E65A and H183A variants were placed under the IPTG‐inducible promoter and introduced into the chromosome of wild‐type FA1090, followed by allelic exchange of *ngo1986* with the kanamycin resistance cassette. Both mutated proteins were stably produced in *N. gonorrhoeae*; however, the H183A variant was present at higher levels than the wild‐type GmhA_GC_ (Figure 4a). Silver staining analysis of LOS revealed that bacteria expressing either E65A or H183A constructs produced truncated LOS, regardless of the presence of IPTG (Figure 4a). Furthermore, under nonpermissive conditions (without IPTG), the ∆*gmhA*_*GC*_/*P*_*lac*_ *::gmhA*_*GC*_E65A and ∆*gmhA*_*GC*_/*P*_*lac*_ *::gmA*_*GC*_H183A, similar to the ∆*gmhA*_*GC*_/*P*_*lac*_ *::gmhA*_*GC*_, had decreased viability, which was demonstrated by a 3214‐, and 8653‐fold decline, respectively, in CFUs in comparison with wild‐type gonococci (Figure 4b). In contrast, the expression of either mutated version of GmhA_GC_ rescued bacterial viability to the wild‐type level.

These *in vivo* studies demonstrated the importance of residues E65 and H183 in GmhA_GC_ activity in the production of full‐length LOS and suggested that abolition of LOS synthesis is disconnected from the GmhA_GC_‐dependent effect on *N. gonorrhoeae* viability.

### 3.6 Transcomplementation studies of GmhA

**Figure 5:**
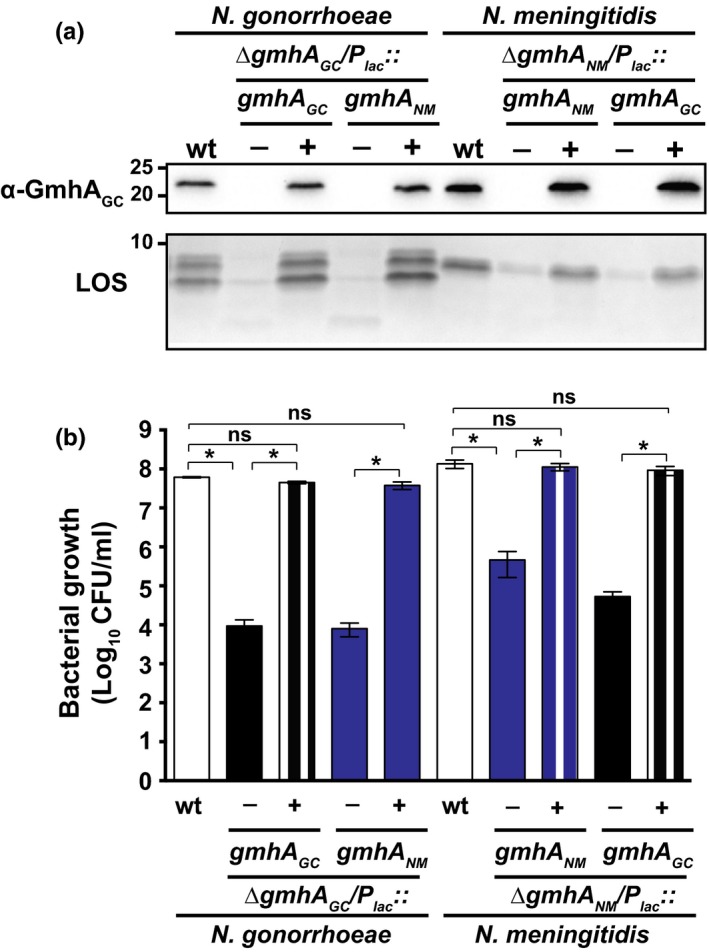
Homologs of GmhA from *N. gonorrhoeae* and *N. meningitidis* function interchangeably. (a) *N. gonorrhoeae* FA1090 wild‐type and isogenic conditional *gmhA* mutants bearing either endogenous (∆*gmh* *A*_*GC*_/*P*_*lac*_ *::gmh* *A*_*GC*_) or *N. meningitidis*‐derived *gmh* *A*_*NM*_ (∆*gmh* *A*_*GC*_/*P*_*lac*_ *::gmh* *A*_*NM*_), as well as *N. meningitidis* MC58 wild‐type and conditional *gmhA* mutants carrying *gmhA* alleles (∆*gmh* *A*_*NM*_/*P*_*lac*_ *::gmh* *A*_*NM*_ or ∆*gmh* *A*_*NM*_/*P*_*lac*_ *::gmh* *A*_*GC*_) were harvested from GCB after 22 hr of incubation either with (+) or without (−) isopropyl β‐D‐1‐thiogalactopyranoside (IPTG). Whole cell lysates were probed with anti‐GmhA_GC_ antibodies or treated with proteinase K and lipooligosaccharide (LOS) was visualized by silver staining. Migration of a molecular mass marker (kDa) is indicated on the left. (b) Wild‐type and mutant strains of *N. gonorrhoeae* and *N. meningitidis,* as described above, were collected from GCB supplemented with 20 μmol/L IPTG and 15 μmol/L IPTG, respectively. Bacteria were suspended in gonococcal base liquid (GCBL) to OD_600_ of 0.1, cultured for 3 hr, back‐diluted to equal OD_600_ of 0.2, serially diluted, and plated on GCB with (+) and without (−) IPTG. CFUs were counted following 22 hr of incubation. Experiments were performed in three independent replicates. Mean values and SEM are presented; *p <* *.05

GmhA homologs of *H. influenzae* and *H. ducreyi* restored the synthesis of full‐length LPS in the *E. coli* ∆*gmhA* mutant (Bauer et al., 1998; Brooke & Valvano, 1996a). This suggested that GmhA proteins can function interchangeably. However, failure of multiple attempts to remove *ngo1986* in FA1090 carrying the *E. coli gmhA* gene cloned under the P_*lac*_ promoter and integrated into the chromosome ruled out the possibility of a functional interspecies complementation. The *E. coli* GmhA (GmhA_EC_) shares 74% and 75% amino acid identity with *H. influenzae* and *H. ducreyi* proteins, respectively, while only 50% identity exists between GmhA_EC_ and GmhA_GC_ (Table 3). In contrast, *N. meningitidis* GmhA (GmhA_NM_) shows 98% identity to GmhA_GC_, and anti‐GmhA_GC_ antisera readily recognized GmhA_NM_ (Figure 5a). Therefore, we decided to use an analogous strategy to create *N. gonorrhoeae* and *N. meningitidis* ∆*gmhA* strains expressing either endogenous or nonendogenous GmhA. Immunoblotting experiments demonstrated that under permissive conditions, both proteins were stably expressed in each host, while without IPTG, neither GmhA_GC_ nor GmhA_NM_ were detected (Figure 5a). Functional interchangeability of GmhA between *N. gonorrhoeae* and *N. meningitidis* was then evaluated by analysis of LOS patterns. In FA1090, ∆*gmhA*_*GC*_/*P*_*lac*_ *::gmhA*_*NM*_ expression of GmhA_NM_ resulted in LOS migrating exactly as LOS species extracted from the wild‐type gonococci (Figure 5a). Likewise, expression of GmhA_GC_ restored LOS synthesis in the *N. meningitidis* MC58 ∆*gmhA*_*NM*_/*P*_*lac*_ *::gmhA*_*GC*_. Furthermore, as expected from our studies in *N. gonorrhoeae* (Figure 1), depletion of GmhA_NM_ in *N. meningitidis* MC58 ∆*gmhA*_*NM*_/*P*_*lac*_ *::gmhA*_*NM*_ had adverse effects on bacterial viability and resulted in a 300‐ and 235.7‐fold decrease in CFUs in comparison with wild‐type and ∆*gmhA*_*NM*_/*P*_*lac*_ *::gmhA*_*NM*_ cultured in the presence of IPTG, respectively (Figure 5b). There was no statistically significant difference in the number of CFUs between the wild‐type *N. gonorrhoeae* and its isogenic ∆*gmhA*_*GC*_ mutant expressing either GmhA_GC_ or GmhA_NM_ (Figure 5b). Similarly, both GmhA_NM_ and GmhA_GC_ fully complemented the lack of GmhA_NM_ in *N. meningitidis*.

Together, the transcomplementation studies showed that the *N. gonorrhoeae* and *N. meningitidis* GmhA can function interchangeably. Expression of either of the homologs restored both viability and LOS synthesis. Additionally, we concluded that *E. coli* GmhA_EC_ is not able to complement GmhA_GC_ functions, as we were not able to generate a viable *N. gonorrhoeae* ∆*gmhA*_*GC*_/*P*_*lac*_ *::gmhA*_*EC*_ mutant.

### 3.7 The structure of *N. gonorrhoeae* GmhA

**Figure 6:**
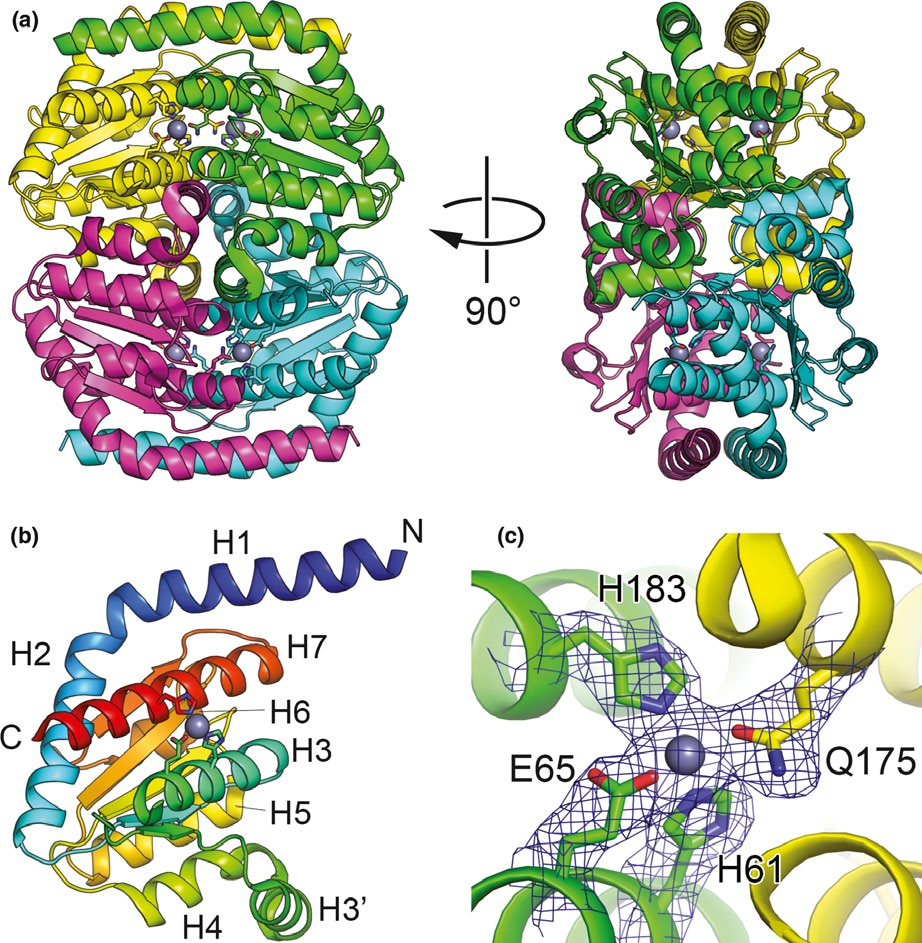
The structure of *N. gonorrhoeae* GmhA_GC_. (a) The ribbon representation of GmhA_GC_ tetramer with monomers colored in green, yellow, cyan, and magenta. Zn^2+^ ions are shown as gray spheres. (b**)** The structure of GmhA_GC_ monomer is colored in rainbow colors from blue (N‐terminus) to red (C‐terminus). The α‐helices are labeled according to Seetharaman et al., 2006. **(**c**)** A close‐up view of the Zn^2+^‐binding site of GmhA_GC_. The coordinating side chains are shown in stick representation. The σ_A_‐weighted 2*F*_O_–*F*_C_ electron density map countered at 1.0 σ is displayed as blue mesh

To gain insights into the function of GmhA_GC_ and to facilitate the future targeting of this enzyme with small molecule inhibitors, we obtained recombinant protein for structural studies (Fig. S1). The structure of *N. gonorrhoeae* GmhA was determined by molecular replacement and was refined to 2.37 Å resolution with *R*_work_ 0.207, *R*_free_ 0.267, and excellent stereochemical parameters (Table 2). Four monomers of GmhA_GC_ were present in the asymmetric unit (Figure 6a). The tetrameric architecture is consistent with the results of size exclusion chromatography (Fig. S1A), and with the previously determined structures of GmhA homologs from other bacteria (Table 3). The interface area of the GmhA_GC_ tetramer is extensive and buries 14,160 Å^2^ of surface area as calculated by the PISA server (Krissinel & Henrick, 2007). Four subunits of GmhA_GC_ adopt highly similar structures with root mean square deviation (r.m.s.d.) 0.1–0.2 Å between subunits. Residues 69–74 (chains A and B) and 69–75 (chains C and D) are disordered in the structure. These residues form a loop in the vicinity of the active site and could become ordered upon substrate binding. A similar disorder was observed in the homologous region of *E. coli* GmhA (Taylor et al., 2008).

**Table 2:** Data collection and refinement statistics

	*N. gonorrhoeae* GmhA_GC_ (PDB 5I01)
Data collection	
Wavelength (Å)	1.0000
Space group	*P*2_1_2_1_2
Cell dimensions:	
*a*,* b*,* c* (Å)	114.36, 130.15, 47.15
α, β, γ (°)	90, 90, 90
Resolution (Å)	85.91–2.37 (2.43–2.37)^a^
*R*_sym_	0.134 (1.032)
CC_1/2_ b	99.6 (62.4)
*I*/σ*I*	9.3 (1.7)
Completeness (%)	95.8 (97.8)
Multiplicity	3.7 (3.6)
Refinement	
Resolution (Å)	85.91–2.37
No. reflections (total/free)	28191/1405
*R*_work_/*R*_free_	0.207/0.267
Number of atoms:	
Protein	5411
Ligand/ion	4
Water	101
*B*‐factors:	
Protein	37.6
Ligand/ion	54.9
Water	32.7
All atoms	37.2
Wilson *B*	41.2
R.m.s. deviations:	
Bond lengths (Å)	0.009
Bond angles (°)	1.224
Ramachandran distribution^c^ (%):	
Favored	97.8
Outliers	0

a Values in parentheses are for the highest resolution shell.

b CC_1/2_ correlation coefficient as defined in (Karplus & Diederichs, 2012) and calculated by *XSCALE* (Kabsch, 2010b).

c Calculated using the MolProbity server (http://molprobity.biochem.duke.edu) (Chen et al., 2010).

**Table 3:** Structural homologs of *N. gonorrhoeae* GmhA_GC_

Protein	Organism	r.m.s.d.^a^	Sequence identity^a^ (%)	Ligands	PDB ID	Reference
GmhA	*P. aeruginosa*	0.5	57	D‐*glycero*‐α‐D‐*manno*‐heptopyranose‐7‐phosphate	1X92	(Taylor et al., 2008)
DiaA	*E. coli*	0.7	52	apo	2YVA	(Keyamura et al., 2007)
DiaA	*E. coli*	0.7	52	apo	4U6N	unpublished
GmhA	*F. tularensis*	0.7	45	apo	3TRJ	(Chaudhury et al., 2013)
GmhA	*C. psychrerythraea*	0.7	48	apo	5BY2	(Do et al., 2015)
GmhA	*C. jejuni*	0.7	54	apo	1TK9	(Seetharaman et al., 2006)
GmhA	*P. aeruginosa*	0.8	56	apo	3BJZ	(Taylor et al., 2008)
GmhA	*B. pseudomallei*	0.8	43	Zn^2+^	2X3Y	(Harmer, 2010)
GmhA	*B. pseudomallei*	0.9	43	Zn^2+^, D‐*glycero*‐α‐D‐*manno*‐heptopyranose‐7‐phosphate	2XBL	(Harmer, 2010)
GmhA	*E. coli*	1.4	45	apo	2I2W	(Taylor et al., 2008)
GmhA	*E. coli*	1.1	45	D‐sedoheptulose‐7‐phosphate	2I22	
GmhA	*V. cholerae*	1.1	43	apo	1X94	(Seetharaman et al., 2006)

a As reported by the Dali server (Holm & Rosenstrom, 2010) for superposition of monomers. A mean value is listed for the structures containing more than one protein subunit in the asymmetric unit.

Each monomer of GmhA_GC_ is composed of a central five‐stranded parallel β‐sheet flanked by four α‐helices from each side (Figure 6b). Four zinc ions are present in the GmhA_GC_ tetramer. The zinc ions are coordinated by the side chains of residues H61, E65, and H183 of one monomer, and the side chain of Q175 of the neighboring monomer (Figure 6c). Therefore, the zinc‐binding sites of GmhA_GC_ are identical to the zinc‐binding sites of *B. pseudomallei* GmhA (Harmer, 2010).

Overall, the structure of GmhA_GC_ is similar to the previously determined structures of GmhA from other bacteria, as well as *E. coli* DiaA (Table 3). The monomer structure of GmhA_GC_ could be superimposed to the homologous structures with r.m.s.d. of 0.5–1.4 Å. The tetramer structure of GmhA_GC_ forms a “closed” conformation, similar to the GmhA structures of *P. aeruginosa*,* V. cholerae*,* B. pseudomallei*, and *C. psychrerythraea* (Do et al., 2015; Harmer, 2010; Seetharaman et al., 2006; Taylor et al., 2008).

## 4 Discussion

GmhA is a conserved sedoheptulose‐7‐phosphate isomerase involved in the first biosynthesis step of the L,D‐heptose component of the LPS/LOS. Deprivation of heptoses results in pleiotropic phenotypes including synthesis of LPS/LOS molecules composed only of lipid A and KDO residues, increased susceptibility to antimicrobial agents, defects in plasmid F conjugations and P1 bacteriophage transduction, and decreased virulence and biofilm formation (Bauer et al., 1998; Brooke & Valvano, 1996a; Earl et al., 2015; Havekes et al., 1976; Malott et al., 2013; Tamaki et al., 1971). In *N. gonorrhoeae*, heptoses are also crucial for LOS function in the evasion of the host immune system and induction of HIV expression (Malott et al., 2013; Preston et al., 1996). Variability in LOS molecules on the gonococcal surface is driven by phase variable enzymes responsible for decorating the sugar moiety of the outer core oligosaccharide (Apicella et al., 1987; Danaher et al., 1995). *N. gonorrhoeae* scavenges sialic acid from the human body and modifies the lacto‐*N*‐neotetraose attached to the HepI of the LOS core oligosaccharide, which provides protection from killing by classical and alternative complement pathways (Elkins et al., 1992; Ram et al., 1998). Thus, inhibition of GmhA could aid in treatment of infections caused by different pathogenic bacteria including *N. gonorrhoeae*.

Accordingly, in this work, we presented initial characterization of GmhA_GC_ at the functional and structural levels. Our experiments demonstrated that gonococci and meningococci depleted in GmhA produced overall significantly less LOS molecules that migrated faster in comparison with the LOS‐derived from wild‐type bacteria (Figures 1b and 5a). In contrast to other orthologs, however, diminution of the GmhA cellular pool had severe consequences on neisserial growth (Figures 1a, d–e and 5b). This protein is likely not essential for bacterial viability, as GmhA*‐*depleted neisserial cells still arose on solid media under nonpermissive conditions (Figure 1a, d–e). Corroborating this observation, *gmhA*_*GC*_ was not found among 827 gonococcal core essential genes identified using a high‐density Tn5 transposon library (Remmele et al., 2014). In addition, expression of GmhA_GC_ variants carrying substitutions in the catalytic residues coordinating zinc ions, E65, and H183 (Figure 6c), led to synthesis of truncated LOS while retaining ample *N. gonorrhoeae* viability (Figure 4). These findings suggested that GmhA_GC_ may be involved in additional physiological function(s) in *Neisseria*. Not surprisingly, a complete functional transcomplementation was achieved between GhmA_GC_ and GmhA_NM_ (Figure 5a–b), whereas multiple attempts to generate a viable strain of *N. gonorrhoeae* expressing only GmhA_EC_ were unsuccessful (data not shown). Based on these findings, we concluded that GmhA plays a critical role in LOS synthesis and is a fundamental growth factor for both *N. gonorrhoeae* and *N. meningitidis*.

Profiling of GmhA_GC_ expression showed that amounts of GmhA_GC_ increased during exponential growth of wild‐type *N. gonorrhoeae* (Figure 2c). Surprisingly, the isogenic conditional ∆*gmhA*_*GC*_/*P*_*lac*_ *::gmhA*_*GC*_ mutant cultured in the presence of IPTG had elevated levels of GmhA_GC_ during the late logarithmic phase (Figure 1c–e), suggesting that expression of GmhA_GC_ is regulated at the posttranscriptional (e.g., small noncoding RNAs, RNA‐binding proteins, RNases, and thermos‐switches) or posttranslational levels. Higher amounts of GmhA_GC_ were also noted upon exposure of *N. gonorrhoeae* to environmental cues relevant to infection; iron deprivation and anaerobiosis (Figure 2d). Transcriptomic studies, however, did not identify GmhA_GC_ as an iron‐regulated gene (Ducey, Carson, Orvis, Stintzi, & Dyer, 2005; Jackson et al., 2010). The observed upregulation of GhmA_GC_ under anaerobic conditions was in agreement with deep sequencing analysis of the *N. gonorrhoeae* anaerobic stimulon, where *gmhA*_*GC*_ was found to be 4.2‐ and 6.1‐fold upregulated in two biological experiments (Isabella & Clark, 2011). Higher levels of enzymes acting downstream from GmhA including HldA (NGO0402) and GmhB (NGO2070) were identified in anaerobically versus aerobically cultured gonococci in our recent high‐throughput proteomic studies (Zielke et al., 2016), suggesting that the L,D‐heptose biosynthesis pathway is upregulated during anoxia. Accordingly, increased amounts of LOS were detected in anaerobically grown gonococci (Figure 2d), while vast overexpression of GhmA_GC_ alone had no effect on LOS levels (Figure 2e). GmhA_GC_ expression was also studied in a diverse collection of gonococcal isolates, including the 2016 WHO reference strains (Unemo et al., 2016) containing multidrug‐resistant *N. gonorrhoeae* (Figure 3). Potential involvement of GmhA in antibiotic resistance via increased synthesis of LPS was suggested recently in *Salmonella typhimurium* DT104B multiresistant strain with additional fluoroquinolone resistance (Correia et al., 2016). Notwithstanding this suggestion, the amounts of GmhA_GC_ varied widely between the WHO strains K, L, V, W, X, Y, and Z, which display overall high levels of ciprofloxacin resistance (MIC>32 mg/L) and decreased susceptibility to gemifloxacin and moxifloxacin (Unemo et al., 2016). Additionally, strain L, which has high MICs for all quinolones (MICs in mg/L for ciprofloxacin, gemifloxacin, and moxifloxacin of >32, 8, and 16, respectively), showed significantly lower GmhA_GC_ levels in comparison with WHO isolates F, O, P, and U, which are all ciprofloxacin sensitive (MIC of 0.004 mg/L) and are significantly more sensitive to gemifloxacin and moxifloxacin (MICs ranging from 0.004 to 0.016 mg/L).

Finally, to better understand the pivotal function of GmhA_GC_, we have determined the three‐dimensional structure of the untagged enzyme (Figure 6). Comparison of our GmhA_GC_ crystal structure with the structures of homologs from other bacteria did not reveal significant differences in the general organization of the structure or within the catalytic site of the enzyme. Zinc ions were not added to any of the protein purification steps or crystallization buffers, yet similar to *Burkholderia pseudomallei* GmhA (Harmer, 2010), GmhA_GC_ held zinc ions in the active site (Figure 6c), providing further support that GmhA is a metalloenzyme. Zinc is likely retained from the cell of the heterologous host during protein overproduction and natively bound to the active site of the enzyme. Zinc binding likely drives the “closed” conformation of GmhA_GC_, which was also observed in structures of *B. pseudomallei*,* P. aeruginosa*, and *V. cholerae* GmhA (Table 3), thus providing further support that the “closed” conformation is catalytically relevant. In addition, site‐directed mutagenesis coupled with *in vivo* studies confirmed the pivotal function of GmhA_GC_ E65 and H183 in the isomerization of the sedoheptulose‐7‐phosphate (Figures 4 and 6).

In conclusion, understanding the function and structure of individual GmhA proteins will facilitate drug discovery approaches focused on targeting this protein with small molecule inhibitors. In particular, our work demonstrated the crucial function of GmhA in neisserial growth and LOS synthesis, positive regulation of expression by host‐relevant environmental stimuli, and conservation among different isolates, as well as provided further support for the mode of action of GmhA. These findings underscore the significance of GmhA_GC_ as a target for antigonorrhea therapeutics. Future work involving determining the interacting partner(s) of GmhA_GC_ and analysis of global changes at the proteome and metabolome levels are required to elucidate the whole scope of physiological function(s) of GmhA_GC_ in *N. gonorrhoeae*.

## Conflict of Interest

The authors have declared that no conflict of interest exist.
