# Supplementary material for: Functional and structural studies on the Neisseria gonorrhoeae GmhA, the first enzyme in the glycero‐manno‐heptose biosynthesis pathways, demonstrate a critical role in lipooligosaccharide synthesis and gonococcal viability
Source: Microbiologyopen. 2017 Jan 7;6(2):e00432. doi: 10.1002/mbo3.432 (PMC5387315; doi:10.1002/mbo3.432)

SUPPLEMENTAL MATERIAL

Supplemental Table S1. Plasmids and oligonucleotides used in this study.

Plasmid Name	Reference
pRSF-NT	(Korotkov *et al.*, 2013)
pGCC4	(Skaar *et al.*, 2002)
pUC18	(Yanisch-Perron *et al.*, 1985)
pUC18K	(Menard *et al.*, 1993)
pRSF-NT-GmhA$_{GC}$	This study
pRSF-NT-Zwf	This study
pGCC4-GmhA$_{GC}$	This study
pUC18K-GmhA$_{GC}$	This study
pUC18-GmhA$_{GC}$	This study
pUC18-GmhA$_{GC}$E65A	This study
pUC18-GmhA$_{GC}$H183A	This study
pGCC4-GmhA$_{GC}$E65A	This study
pGCC4-GmhA$_{GC}$H183A	This study
pGCC4-GmhA$_{NM}$	This study
pUC18K-GmhA$_{NM}$	This study
pGCC4-GmhA$_{EC}$	This study
**Oligonucleotide**	**Sequence[1]**
Primers used for creating recombinant protein with N-terminal 6×His-tag:	
rNGO1986-F	GGATTTACCCATGGCGACATTACAAGAACGCG
rNGO1986-R	ACTCGGTCAAGCTTCATTCCTTCCAGCAGTACG
rZwf-F	GACTCCATGGGTACACAGACAAATTTTGATTTGG
rZwf-R	GATCAAGCTTGCATTACTGTTCTTCGTGC
Primers used for gene deletion and complementation:	
NGO1986-Up-F	GACTGATAGAATTCGCGCAGGGTAATGTCTG
NGO1986-Up-R	ATCGATGGTACCGCAACGCGTTCTTGTAATG
NGO1986-Down-F	GGATTTACGGATCCACTGTATCGACTCCGTACTGC
NGO1986-Down-R	ACTCGGTCAAGCTTTGATGCCCAGCAGCGTG
NGO1986-RBS-F	GAATATTACAGGTTGACGATATG
NGO1986-RBS-R	AAGCTTGGCCGGCCTTACATTCCTTCCAGCAG
pGCC4-Ver-F	AAATCGCCCTTGATACCG
pGCC4-Ver-R	ACACTTTATGCTTCCGGCTC
NGO1986-Ver-F	GCCGGCGTACCCGCAT

NGO1986-Ver-R	TGAAGGCGGTTCAGACGGC
NMB2090-Up-F	GACTGATAGAATTCCAATACCGCCAAAGCG
NMB2090-Down-R	ACTCGGTCAAGCTTTGATGCCCAACAGCGTG
ECBD3400-RBS-F	TTATGCTGAAGGATATCCTC
ECBD3400-RBS-R	AAGCTTGGCCGGCCTTACTTAACCATCTCTTTTTCAATC
Primers used for the mutagenesis of the GmhA$_{GC}$ conserved residues E65 and H183:	
E65A-F	CTTCGCCGCCGCAATGACCGGGC
E65A-R	TGTTGCGCGTCGGCAGCC
H183A-F	CCTGCTGATAGCCGCCATGTGCG
H138A-R	ATGTGGTTTTCCTGAATGC
Primers used for the sequencing of lactose operator and promoter:	
LACOP-F	TCCCTTAACTTGTTTTTCGTGTACC
LACOP-R	CGCTTACCCTTCCTGAAGACA
LACOP-SEQ	CCGACATCATAACGGTTCTGGC

[1] Sequences recognized by restriction enzymes are underlined.

Supplemental Figure Legends

Supplemental Figure S1. Purification of rGmhA$_{GC}$. A. rGmhA$_{GC}$ eluted mainly as tetramers during size exclusion chromatography. Purified rGmhA$_{GC}$ was separated by NGC Scout Chromatography system (Bio-Rad) with HiLoad 16/600 Superdex 75 pg column (GE Healthcare Life Sciences). Elution chromatogram of rGmhA$_{GC}$ is indicated by blue line. Predicted mass of a monomeric rGmhA$_{GC}$ is 21.1 kDa. Gel Filtration Standard (BioRad) is shown on the chromatogram as the red line. **B.** Increasing amounts of rGmhAGC after removal of His-tag by TEV protease were resolved by SDS-PAGE and visualized by Coomasie Brilliant Blue G-250 staining. The migration of molecular mass markers (kDa) is indicated on the left.

Supplemental Figure S2. The *Neisseria gonorrhoeae* FA1090 Δ*gmhA*$_{GC}$/P$_{lac}$::*gmhA*$_{GC}$ acquires mutations in *P*$_{lac}$ during growth under non-permissive conditions. A. The *N. gonorrhoeae* FA1090 Δ*gmhA*$_{GC}$/*P*$_{lac}$::*gmhA*$_{GC}$, was streaked out from frozen glycerol stock on solid media supplemented with 20 µM IPTG. Following 18 h of incubation at 37 °C in the presence of 5% atmospheric CO_2, the non-piliated colonies were passaged onto plates either with (+) or without IPTG (-). Bacterial colonies that arose on media without IPTG (-) were passaged (passage II) further onto plates either with (+) or without IPTG (-) and incubated for 18 h at 37 °C, 5% CO_2. Colonies that grew on media lacking IPTG were once more streaked (passage III) onto plates either with (+) or without IPTG (-) and allowed to grow for 18 h at 37 °C, 5% CO_2. **B.** The lactose promoter (*P*$_{lac}$) sequence of the *gmhA*$_{GC}$/*P*$_{lac}$::*gmhA*$_{GC}$ maintained in the presence of IPTG and after three passages on media without IPTG were determined by DNA Sanger sequencing. The sequence of *P*$_{lac}$ from *E. coli* is shown. Nucleotides that differed from the default *P*$_{lac}$ sequence are highlighted in red color. **C.** The FA1090 wt, Δ*gmhA*$_{GC}$/*P*$_{lac}$::*gmhA*$_{GC}$, and Δ*gmhA*$_{GC}$/*P*$_{lac}$::*gmhA*$_{GC}$ after three passages on media without IPTG were harvested from solid media with (+) or without 20 µM IPTG (-) and whole cell lysates were either probed with polyclonal rabbit antisera or subjected to LOS extraction using proteinase K followed by silver staining. Samples were matched by the same OD_{600} units. The migration of molecular mass marker (kDa) is indicated on the left.

Supplemental Figure S3. Loading controls for immunoblotting experiments. Samples of whole-cell lysates were prepared for SDS-PAGE as described in the text, separated in 10-20% Tris-Glycine gel and the protein profiles were visualized using

Coomasie Brilliant Blue G-250. Loaded OD_{600} units matched the corresponding samples used in immunoblotting analyses of $GmhA_{GC}$ and Zwf. The migration of molecular mass markers (kDa) is indicated on the left.

Supplemental Figure S4. Analysis of single nucleotide polymorphisms in *gmhA.* Analysis of *gmhA* (locus NGO1986, NMB2090, NMC2070) in 39,182 *Neisseria spp* genomes deposited into PubMLST database (http://pubmlst.org/neisseria/ as of July, 20, 2016) showed that there are 340 *gmhA* alleles and 323 single nucleotide polymorphic sites.

REFERENCES

Korotkov, K.V., J.R. Delarosa & W.G. Hol, (2013) A dodecameric ring-like structure of the N0 domain of the type II secretin from enterotoxigenic *Escherichia coli. Journal of structural biology* **183**: 354-362.

Menard, R., P.J. Sansonetti & C. Parsot, (1993) Nonpolar mutagenesis of the ipa genes defines *IpaB*, *IpaC*, and *IpaD* as effectors of *Shigella flexneri* entry into epithelial cells. *Journal of Bacteriology* **175**: 5899-5906.

Skaar, E.P., M.P. Lazio & H.S. Seifert, (2002) Roles of the *recJ* and *recN* genes in homologous recombination and DNA repair pathways of *Neisseria gonorrhoeae*. *Journal of Bacteriology* **184**: 919-927.

Yanisch-Perron, C., J. Vieira & J. Messing, (1985) Improved M13 phage cloning vectors and host strains: nucleotide sequences of the M13mp18 and pUC19 vectors. *Gene* **33**: 103-119.

Supplemental Figure S1

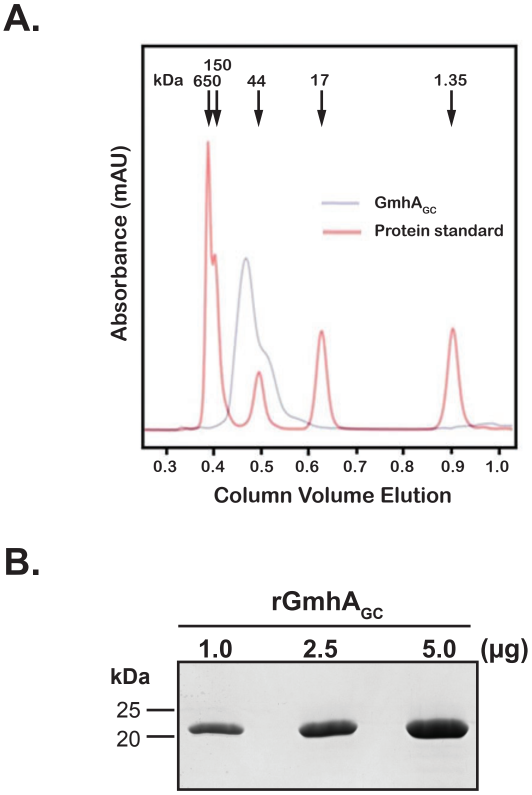

Supplemental Figure S2

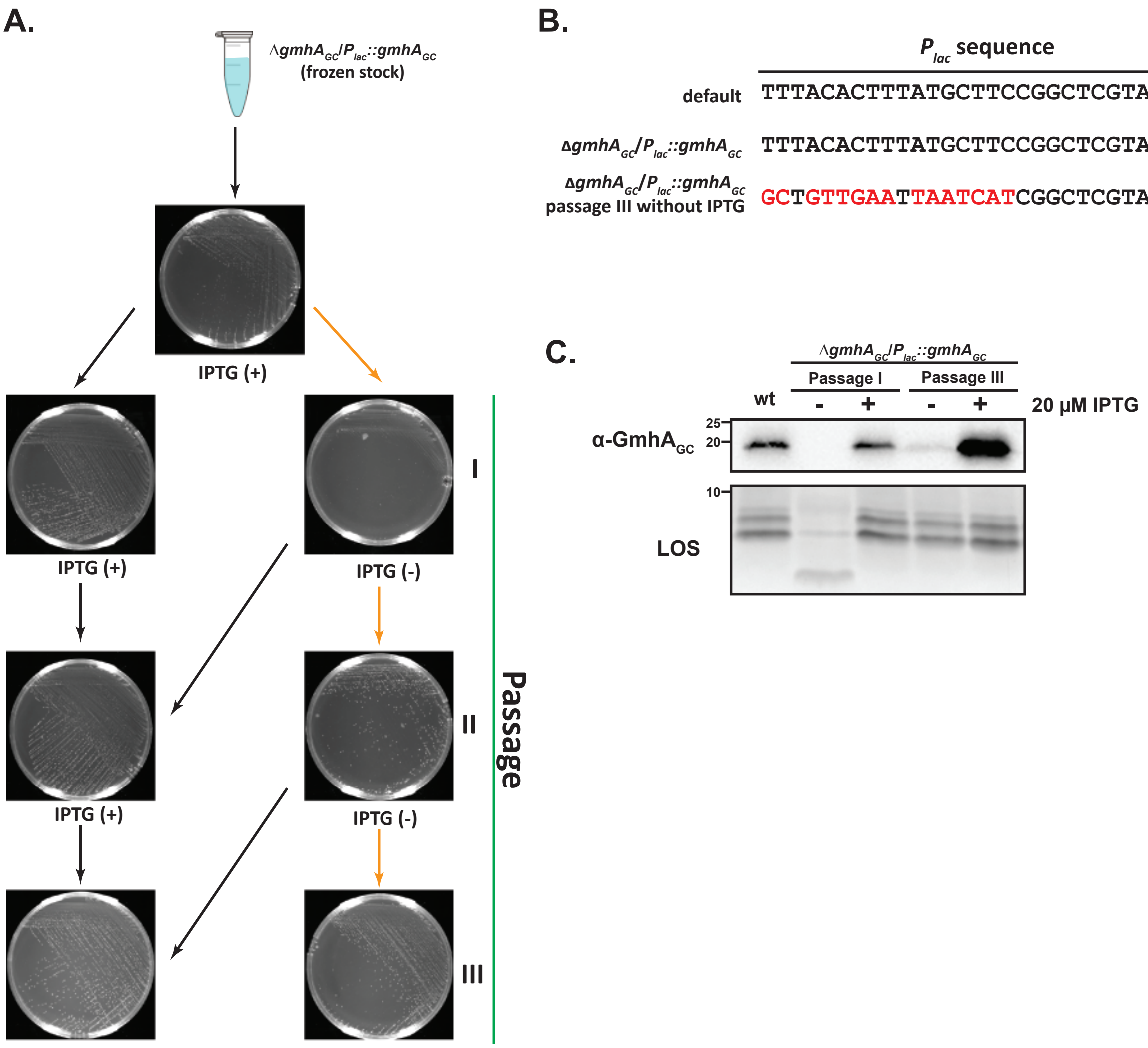

Supplemental Figure S3

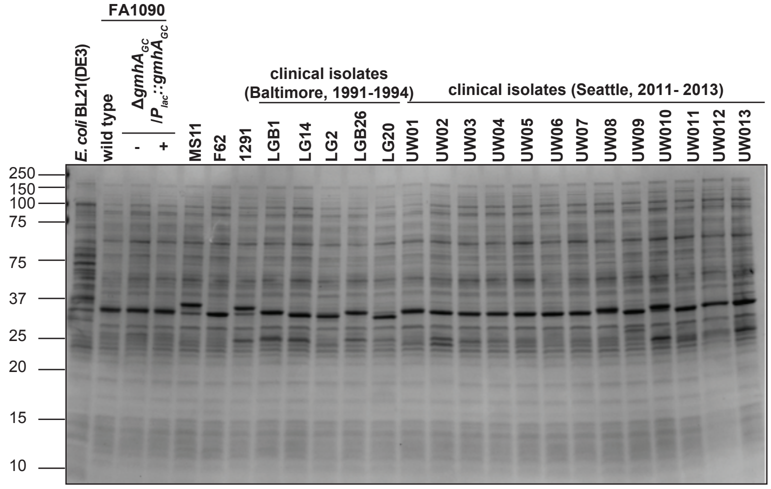

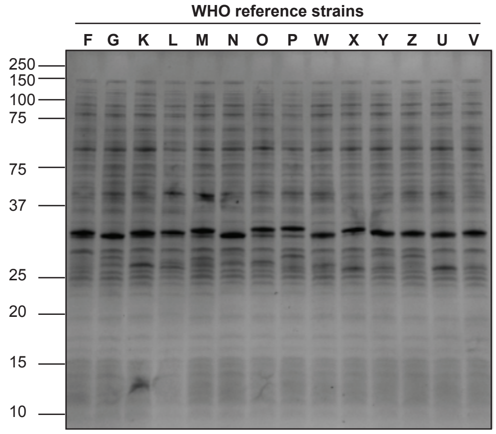

Supplemental Figure S4

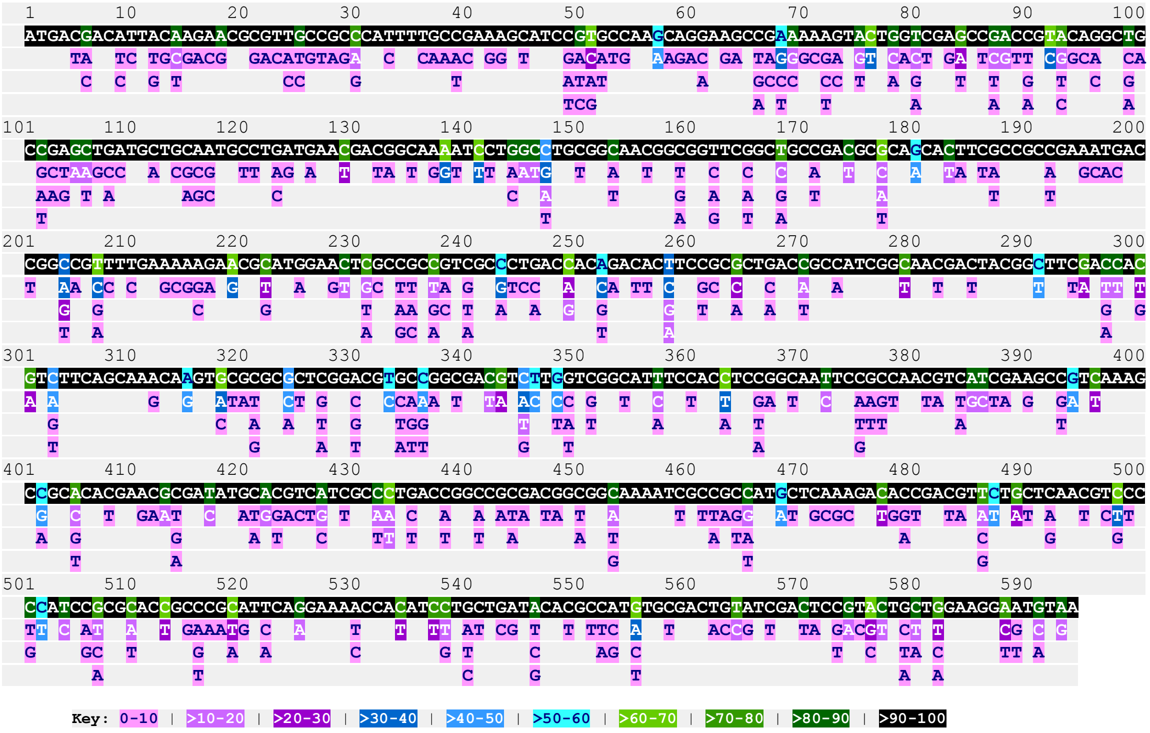

Supplement: Supplementary file 1 [file MBO3-6-na-s001.pdf]
